# Optimization of technological procedure for amygdalin isolation from plum seeds (*Pruni domesticae semen*)

**DOI:** 10.3389/fpls.2015.00276

**Published:** 2015-04-28

**Authors:** Ivan M. Savic, Vesna D. Nikolic, Ivana M. Savic-Gajic, Ljubisa B. Nikolic, Svetlana R. Ibric, Dragoljub G. Gajic

**Affiliations:** ^1^Faculty of Technology, University of NisLeskovac, Serbia; ^2^Faculty of Pharmacy, University of BelgradeBelgrade, Serbia; ^3^School of Electrical Engineering, University of BelgradeBelgrade, Serbia; ^4^Center of Excellence DEWS, University of L'AquilaL'Aquila, Italy

**Keywords:** amygdalin, plum seeds, extraction, central composite design, multilayer perceptron, isolation, structural characterization

## Abstract

The process of amygdalin extraction from plum seeds was optimized using central composite design (CCD) and multilayer perceptron (MLP). The effect of time, ethanol concentration, solid-to-liquid ratio, and temperature on the amygdalin content in the extracts was estimated using both mathematical models. The MLP 4-3-1 with exponential function in hidden layer and linear function in output layer was used for describing the extraction process. MLP model was more superior compared with CCD model due to better prediction ability. According to MLP model, the suggested optimal conditions are: time of 120 min, 100% (v/v) ethanol, solid-to liquid ratio of 1:25 (m/v) and temperature of 34.4°C. The predicted value of amygdalin content in the dried extract (25.42 g per 100 g) at these conditions was experimentally confirmed (25.30 g per 100 g of dried extract). Amygdalin (>90%) was isolated from the complex extraction mixture and structurally characterized by FT-IR, UV, and MS methods.

## Introduction

Amygdalin presents one of natural cyanogenic glycosides that is contained in the seed of apricot, apple, black cherry, peach, plum, etc… (Bolarinwa et al., 2014). The chemical structure of amygdalin is presented in Supplementary Figure S.1. The anti-tumor activity of amygdalin can be enhanced due to its hydrolysis after the action of β-glucosidase enzyme and release of hydrocyanic acid (Zhou et al., 2012).

The traditional optimization technique, i.e., a one-variable-at-a-time (OVAT) method implies the change of one variable at a time instead of all simultaneously (Kokosa et al., 2009). This approach requires more experimental runs for the same precision in the effect estimation. Also, such a method cannot estimate the interactions between process parameters and commonly leads to wrong optimal conditions. Because of these reasons, the central composite design (CCD) increasingly has a wide application in modeling of the observed processes. Actually, CCD represents an experimental design, which belongs to the response surface methodology (RSM). The use of CCD requires smaller number of performed experiments compared with the OVAT approach, so that the consumption of all resources such as chemicals and time is reduced.

Kassama et al. (2008) used this model to optimize the parameters for the supercritical carbon dioxide extraction of lycopene (C_40_H_56_) from dried tomato skins. The ultrasound-assisted extraction of important functional components from Campbell Early grape seed was carried out according to a five-level, three-variable CCD (Ghafoor et al., 2009). CCD was used to investigate experimental process conditions for the supercritical ethanol extraction of bio-oils from German beech wood (Akalin et al., 2013). The microwave extraction of polyphenols from grape residues in presence of Na_2_CO_3_ was also modeled using CCD (Brahim et al., 2014). Amyrgialaki et al. (2014) used CCD for optimization of the extraction of pomegranate (*Punica granatum*) husk phenols using water/ethanol solvent systems. Also, Chua et al. (2009) optimized the ultrasound extraction of phospholipids from the palm-pressed fiber.

Artificial neural networks (ANNs) inspired by biological neural networks are used for modeling of nonlinear and complex systems (Mehrotra et al., 1997). ANNs are very useful for finding a relationship between input and output variables. Actually, the inputs of ANNs correspond to the dendrites, while an output represents the axon in biological neural networks. An artificial neuron receives one or more inputs and sums them up to generate an output. In recent years, ANNs play a significant role in optimization of different processes, such as adsorption (Ebrahimzadeh et al., 2012; Savic et al., 2012b, 2014) and extraction (Turan et al., 2011; Khajeh et al., 2012; Sinha et al., 2012; Savic et al., 2013). One of important feedforward ANN is a multilayer perceptron (MLP). The MLP is commonly presented as a superior mathematical approach in response prediction compared with CCD model (Savic et al., 2012b, 2013). This model consists from three layers (input, hidden, and output layers). Savic et al. (2013) optimized the extraction procedure of the total flavonoids from green tea. They used three process parameters in order to model and obtain optimal conditions for extraction of bioactive compounds. Sinha et al. (2012) modeled the microwave-assisted natural dye extraction from pomegranate rind using three-layer feedforward backpropagation neural network with a linear transfer function. A multilayer feed-forward neural network trained with an error back-propagation algorithm was also employed for developing a model for prediction of the yield of essential oils from *Diplotaenia cachrydifolia* by supercritical fluid extraction (Khajeh et al., 2012). The key difference between these mathematical models is the polynomial equation, which is explicitly obtained only by using CCD model.

Amygdalin was initially isolated from the seeds of bitter almond (*Prunus dulcis*) by French chemists in 1830 (Song and Xu, 2014). The procedures for amygdalin isolation from apricot (*Prunus armeniaca*) (Lv et al., 2005; Yan et al., 2006; Muhammad and Abbas, 2013) and colocynth (*Citrullus colocynthis*) (Muhammad and Abbas, 2013) were described in the literature, but there is no data about the use of plum seeds as the plant material. Thus, the aim of this paper was to model and optimize the extraction of amygdalin from this plant material using both CCD and MLP models, as well as to isolate amygdalin from the complex extraction mixture using diethyl ether. Different methods were used in order to confirm the structure of the isolated amygdalin.

## Materials and methods

### Reagents

Amygdalin standard purity ≥97%, potassium bromide spectroscopic purity (Sigma-Aldrich, Munich, Germany), acetonitrile HPLC grade (Merck, Darmstadt, Germany), methanol LC-MS purity (Avantor Performance Materials, Inc., Deventer, Netherlands), formic acid purity 99% (Carlo Erba Reagents, Val de Reuil, France), ethanol 96% (v/v) (Zorka Pharma, Šabac, Serbia), absolute ethanol (Alkaloid AD, Skopje, Republic of Macedonia), diethyl ether purity ≥99% (VWR International, Lutterworth, UK).

### Plant material

The fresh plum seeds (*Pruni domesticae semen*) of Stanley sort were purchased from PD Plemic komerc (Osečina, Serbia). Firstly, the plant material was dried to the moisture content of 6% in a dark place and then ground in a blender to obtain a fine powder with average particle size of 0.3 mm.

### Extraction and isolation procedure

Plant material (2 g) was transferred into the round bottom flask of 100 cm^3^ and treated with exactly defined volume of ethanol. The extractions at higher temperatures were carried out under reflux, while the extractions at lower temperatures were carried out in closed vessels. The system temperature was maintained using a water bath. After extraction, the extract was separated from the solid plant material by filtering process. Plant extracts were evaporated in rotary evaporator under reduced pressure to remove the solvent, and then stored in desiccator until it reached the constant mass. After addition of diethyl ether (10 cm^3^) to the complex extraction mixture, amygdalin was precipitated, while fatty compounds were dissolved. The dissolved fatty compounds were removed by decanting process. The solid phase was dried at 30°C in order to remove the residual solvent. In this way, the sample was prepared for determination of its purity using HPLC analysis and structural characterization by FT-IR, UV, and MS methods.

### Modeling and optimization of extraction procedure

#### Central composite design

The effective extraction parameters, such as extraction time, ethanol concentration, solid-to-liquid ratio and extraction temperature were estimated and optimized using CCD. Statistica 8.0 (Stat Soft, Inc., Tulsa, USA) software was used to analyze the experimental results and build the regression model, which helped to predict the optimal process parameters. For statistical calculations, the variables *X_i_* (the uncoded value of the independent variable) were coded as *x_i_* (the coded value of the independent variable) according to the following equation:

(1) $$x_{i}=\frac{X_{i}-X_{o}}{\Delta X_{i}}$$

where *X_o_* is the value of *X_i_* at the center point, and Δ*X_i_* is the step change value.

The input variables and their levels in terms of coded and actual values are shown in Table 1.

**Table 1:** **Experimental range and levels of the input variables used in the CCD model in terms of actual and coded factors**.

**Independent variables**	**Uncoded**	**Coded**	**Values of coded levels**				
			**−2**	**−1**	**0**	**+1**	**+2**
Time, min	*X*_1_	*x*_1_	10	37.5	65	92.5	120
Ethanol concentration, %	*X*_2_	*x*_2_	20	40	60	80	100
Solid-to-liquid ratio, m/v	*X*_3_	*x*_3_	1:5	1:10	1:15	1:20	1:25
Temperature, °C	*X*_4_	*x*_4_	22	36	50	64	78

In accordance with the CCD matrix, 30 experiments were carried out for building a quadratic model. The sets of 4^2^, 2 × 4 and 6 experimental runs correspond to the points of factorial design, axial points and center points, respectively. The center points was replicated six times in order to estimate experimental errors. The general form of quadratic equation can be presented in the following way (Equation 2):

(2) $$Y=\beta_{o}+\sum_{i\;=\;1}^{k}\beta_{i}x_{i}+\sum_{i\;=\;1}^{k}\beta_{ii}x_{i}^{2}+\sum_{i\;=\;1}^{k}\sum_{i\;\neq \;j=\;1}^{k}\beta_{ij}x_{i}x_{j}+\varepsilon$$

where *x_i_*, *x_j_* are the input variables, β_*o*_ is intercept, β_*i*_, β_*ii*_, β_*ij*_ are the first-order, quadratic, and interaction coefficients, respectively, *i* and *j* are the index numbers for the factors, ε is the residual error, *Y* is the response function.

#### Artificial neural network

ANN was used to provide a nonlinear mapping between input variables (extraction time, ethanol concentration, solid-to-liquid ratio, and temperature) and the output variable (the amygdalin content per 100 g of the dried extract). ANN has been applied to simulate the same experimental data as used for CCD. Statistica 8.0 (Stat Soft, Inc., Tulsa, USA) software was also used for building the neural network models. MLP network was chosen as a powerful network for modeling and optimization. Commonly a feed forward architecture of ANN is used to build a predictive model. In this model, data always flows forward, i.e., from input to output layer. A weight presents a connection between two neurons, which is an adjustable parameter of the network. The neurons in the input layer introduce the scaled input data to the hidden layer via weights. The role of neurons in the hidden layer is to sum up the weighted inputs, including a bias (internal input), in the following way (Equation 3):

(3) $$sum=\sum_{i=1}^{n}x_{i}w_{i}+b$$

where *w_i_* are the weights, *b* is the bias and *x_i_* is the input parameter.

After that, the weighted output is passed through an activation function. In this way, the output of the hidden layer presents the input of the output layer. The neurons in the output layer have the same role as the neurons in the hidden layer. During training of ANNs, an error function is minimized by adjusting the adequate weights in order to obtain approximately the same values of calculated output and actual experimental output.

In this study, the adequacy of fitting the experimental data was estimated based on coefficient of determination (*R*^2^), cross validation correlation coefficient (*Q*^2^), root mean square error (RMSE), mean square error (MSE), and mean absolute error (MAE) for the tested models. These statistical parameters are calculated in the following way (Equations 4–7):

(4) $$Q^{2}=1-\frac{\sum_{i\;=\;1}^{n}{\left(y_{i}^{m}-y_{i}^{p}\right)}^{2}}{\sum_{i\;=\;1}^{n}{\left(y_{i}^{p}-\bar{y}\right)}^{2}}$$

(5) $$RMSE=\sqrt{\frac{\sum {\left(y_{i}^{p}-y_{i}^{m}\right)}^{2}}{n}}$$

(6) $$MSE=\frac{\sum {\left(y_{i}^{p}-y_{i}^{m}\right)}^{2}}{n}$$

(7) $$MAE=\frac{\left|y_{i}^{p}-y_{i}^{m}\right|}{n}$$

where *y^p^_i_* is the desired output, *y^m^_i_* is the actual output, *y* is the average observed value, and *n* is number of experimental runs.

Optimization of the prediction model was performed by application of simplex algorithm in order to maximize the content of amygdalin in the dried extract.

#### HPLC analysis

RP-HPLC method described by Savic et al. (2012a) was used for the analysis of amygdalin in the plum seeds extract. The used mobile phase was water/acetonitrile in the ratio of 25:75 (v/v). The flow rate of mobile phase with isocratic elution was 0.9 cm^3^ min^−1^. Before analysis, the solutions were filtered through a 0.45 μm millipore filter (Econofilters, Agilent Technologies, Germany). The injected volume of samples was 20 μL. The quantification was effected by measuring at 215 nm. The separation was performed at the temperature of 25°C and using a SUPELCO Analytical HS-C18 column (4.6 × 250 mm, 5 μm). The analysis of samples was performed on the Agilent 1100-Series HPLC system consisting of Agilent 1100-Series DAD detector and Agilent 1100-Series auto-sampler (Faculty of Technology, Leskovac).

#### Samples preparation for HPLC analysis

In order to define the content of amygdalin in the dried extract and the purity of amygdalin after ether washing the ethanol extract samples (125 mg) were put into the flask of 10 cm^3^ and then filled by a mobile phase to the mark. After a 5-min sonication, the solution (1 cm^3^) was transferred to the flask of 10 cm^3^ and diluted by the mobile phase. The sample was filtered through a cellulose membrane filter (0.45 μm, Econofilters, Agilent Technologies, Germany) and aliquot of 20 μL was injected into HPLC system. The identification of amygdalin in the plum seeds extract and in the isolate was performed based on comparison of retention times and UV spectra with amygdalin standard.

#### FT-IR analysis of isolated amygdalin

IR spectra were recorded on Bomem MB-100 (Hartmann and Braun) FTIR spectrometer with standard DTGS/KBr detector and scanning in the wavenumber range of 4000–400 cm^−1^ at the resolution of 2 cm^−1^. A potassium bromide disk technique was used for preparation of samples. The sample of 1 mg was homogenized with 150 mg of KBr. The solid mixture was vacuumed and pressed into a pellet.

## Results and discussion

### Optimization of extraction procedure

Solubility of amygdalin in water and ethanol is 83 g dm^−3^ and 1 g dm^−3^, respectively. Amygdalin in water is decomposed into benzaldehyde, hydrocyanic acid, and glucose after the effect of hydrolytic enzyme of emulsin (Hwang et al., 2002a). Amygdalin epimerization is occurred in boiling water (Hwang et al., 2002b), and especially in alkaline medium due to weak acidic character of benzyl proton (Kang et al., 2000). In this study, ethanol was used as a suitable solvent for amygdalin extraction from plum seeds. The extraction temperature increases the yield of extracted compounds due to the reduction of the system viscosity and mass transfer resistance. Lower extraction temperatures are used in the case of thermolabile compounds and when there is a possibility to transform one epimeric form into another. Having in mind that amygdalin tends to epimerize at higher temperatures, the extractions were performed at the temperatures lower than 100°C. Also, the limiting temperature for modeling the extraction using the CCD and MLP models was the boiling temperature of absolute ethanol, i.e., 78°C.

Identification and quantification of amygdalin in the plant extract was performed using the developed and validated HPLC method. The content of amygdalin was calculated and expressed per 100 g of the dried extract.

### Central composite design

The effect of extraction time (10–120 min), ethanol cocnentration (20–100%, v/v), solid-to-liquid ratio (1:5–1:25, m/v) and extraction temperature (22–78°C) on the content of amygdalin in the dried extract was studied using CCD with four variables. The experimental data for the content of amygdalin in the extracts (Table 2) were approximated by a quadratic function. After that, the efficiency of models for prediction of amygdalin content in the plant extract was estimated based on the error parameters and correlation coefficients.

**Table 2:** **Central composite design matrix of independent variables and their corresponding experimental and predicted values of the amygdalin content in the extracts**.

**Exp. No. ANN**	**Std CCD**	τ **(min)**	**Ce(%)**	**ω (m/v)**	***t* (°C)**	**Y_obs_(g/100 g d.e.)**	**CCD**	**MLP**
							**Y_pred_**	**Y_pred_**
							**(g/100 g d.e.)**	
1^train^	24	65.0	60	1:15	78	6.51	6.45	6.26
2^train^	7	37.5	80	1:20	36	10.38	10.50	10.58
3^train^	16	92.5	80	1:20	64	12.29	12.26	12.33
4^train^	17	10.0	60	1:15	50	6.85	6.47	6.54
5^train^	19	65.0	20	1:15	50	7.83	7.55	7.64
6^train^	6	92.5	40	1:20	36	11.57	11.22	11.20
7^train^	22	65.0	60	1:25	50	11.09	10.64	10.67
8^train^	11	37.5	80	1:10	64	4.15	4.80	4.84
9^train^	8	92.5	80	1:20	36	14.83	14.93	14.77
10^train^	30 (C)	65.0	60	1:15	50	8.35	8.50	8.15
10^train^	27 (C)	65	60	1:15	50	8.10	8.50	8.15
11^train^	1	37.5	40	1:10	36	9.35	9.27	9.43
12^train^	2	92.5	40	1:10	36	9.65	9.97	10.0
13^train^	3	37.5	80	1:10	36	9.30	8.92	9.30
10^train^	29 (C)	65.0	60	1:15	50	8.42	8.50	8.15
14^train^	13	37.5	40	1:20	64	6.82	6.91	6.77
10^train^	28 (C)	65.0	60	1:15	50	8.26	8.50	8.15
15^train^	5	37.5	40	1:20	36	9.15	9.58	9.48
16^train^	23	65.0	60	1:15	22	13.37	13.24	13.23
17^train^	14	92.5	40	1:20	64	8.27	8.55	8.91
10^train^	26 (C)	65.0	60	1:15	50	8.88	8.50	8.15
18^train^	9	37.5	40	1:10	64	4.94	5.15	4.89
19^train^	10	92.5	40	1:10	64	5.99	5.85	6.23
20^train^	18	120.0	60	1:15	50	11.41	11.59	11.73
21^train^	21	65.0	60	1:5	50	6.66	6.36	6.34
22^train^	12	92.5	80	1:10	64	8.92	8.29	8.26
23^train^	15	37.5	80	1:20	64	7.93	7.83	7.87
24^train^	20	65.0	100	1:15	50	10.82	10.91	11.09
10^train^	25 (C)	65.0	60	1:15	50	8.45	8.50	8.15
25^train^	4	92.5	80	1:10	36	12.10	12.40	12.08

*τ, extraction time; Ce, ethanol concentration; ω, solid-to-liquid ratio; t, extraction temperature; Y_obs_, observed value, Y_pred_, predicted value, d.e., dried extract; Std, standard order*.

The results of analysis of variance (ANOVA) are given in Table 3. The statistical significance of model and equation terms were estimated based on the *p*-values. The *p*-values lower than 0.05 indicate the statistically significant terms at the 95% confidence level. The insignificant terms were excluded from the quadratic model in order to obtain the reduced polynomial model. The *F*-value of the reduced model (105.04) is higher than the critical *F*_(0.05,11,18)_ = 2.37, meaning that model was adequate for prediction of the amygdalin content in the dried extract. In addition, the *F*-value of lack of fit (2.55) was not significant relative to the pure error (0.34), because its value was lower than the critical *F*-value of 4.66. Actually, there is a 15.40% chance that the *F*-value of lack of fit could occur due to noise. *R*^2^_pred_of 0.9520 is in reasonable agreement with *R*^2^_adj_ of 0.9753, while *R*^2^ of 0.9847 indicates that 98.47% of the variation in the amygdalin content in the extract can be explained using this regression model. Also, the parameter of adequate precision is used in order to evaluate the signal-to-noise ratio, whose value should be higher than 4. In this case, the adequate precision of 41.85 implies that this model can be used to navigate the design space.

**Table 3:** **Analysis of variance (ANOVA) for the experimental results of the CCD model**.

	**SS**	**df**	**MS**	***F***	***p***
Model	169.13	11	15.38	105.04	<0.0001
X_1_	39.32	1	39.32	268.64	<0.0001
X^2^_1_	0.49	1	0.49	3.37	0.0831
X_2_	16.90	1	16.90	115.46	<0.0001
X^2^_2_	0.92	1	0.92	6.30	0.0219
X_3_	27.52	1	27.52	188.02	<0.0001
X_4_	69.16	1	69.16	472.47	<0.0001
X^2^_4_	3.15	1	3.15	21.49	0.0002
X_1_ X_2_	7.78	1	7.78	53.18	<0.0001
X_1_ X_3_	0.88	1	0.88	6.04	0.0244
X_2_ X_3_	1.61	1	1.61	11.02	0.0038
X_3_ X_4_	2.09	1	2.09	14.27	0.0014
Residual	2.63	18	0.15		
Lack of fit	2.29	13	0.18	2.55	0.1540
Pure error	0.34	5	0.07		
Total SS	171.76	29			

*SS, sum of squares; df, degree of freedom; MS, mean square; X_1_, extraction time; X_2_, ethanol concentration; X_3_, solid-to-liquid ratio; X_4_, extraction temperature*.

Pareto chart of standardized effects for the reduced polynomial model is presented in Supplementary Figure S.2. The effects are arranged in descending order of significance. All process variables have the significant impact on the amygdalin content in the extracts out of which the temperature has the highest and negative effect. Thus, the content of amygdalin in the extracts decreases when the extraction temperature increases. After the temperature, the extraction time and the solid-to-liquid ratio have a significant contribution to increased content of amygdalin in the extracts. The lowest effect has the ethanol concentration, because the *p*-value for this term of equation is lower than 0.05. In addition to the linear terms, the interactions *x*_1_*x*_2_, *x*_3_*x*_4_, *x*_2_*x*_3_, *x*_1_*x*_3_ and the quadratic terms *x*_42_ and *x*_22_ are also statistically significant terms.

The reduced polynomial model in terms of coded variables can be presented in the following way (Equation 8):

(8) $$\begin{array}{l} Y=\;8.502+1.280x_{1}+0.133x_{1}^{2}+0.839x_{2}+0.181x_{2}^{2}\\ \text{ Full Text Full Text Full Text Full Text Full Text Full Text }+1.071x_{3}-1.698x_{4}+0.335x_{4}^{2}+0.698x_{1}x_{2}+0.235x_{1}x_{3}\\ \text{ Full Text Full Text Full Text Full Text Full Text Full Text }+0.318x_{2}x_{3}+0.361x_{3}x_{4} \end{array}$$

The empirical equation obtained after replacing the coded values with the actual values can be presented as (Equation 9):

(9) $$\begin{array}{l} Y=\;25.799-0.078X_{1}+1.84\cdot 10^{-4}X_{1}^{2}-0.143X_{2}\\ \text{ Full Text Full Text Full Text Full Text Full Text Full Text Full Text }+4.5\cdot 10^{-4}X_{2}^{2}-0.345X_{3}-0.370X_{4}+1.71\cdot 10^{-3}X_{4}^{2}\\ \text{ Full Text Full Text Full Text Full Text Full Text Full Text Full Text }+1.27\cdot 10^{-3}X_{1}X_{2}+1.71\cdot 10^{-3}X_{1}X_{3}+3.17\cdot 10^{-3}X_{2}X_{3}\\ \text{ Full Text Full Text Full Text Full Text Full Text Full Text Full Text }+5.16\cdot 10^{-3}X_{3}X_{4} \end{array}$$

Normal probability plot of the standardized residuals is presented in Supplementary Figure S.3. The standardized residual of the regression model was used in order to define whether the error term ε is actually normally distributed. It can be concluded that the residuals follow the normal distribution due to small deviations from the straight line.

### Multilayer perceptron

After application of CCD for modeling of amygdalin extraction, additional set of experimental data for testing and validation of ANN was generated randomly. These combination of process variables were within the limits of CCD, i.e., in the range of −α < *x* < +α and given in Table 4. Actually, validation set is used for tuning of the model parameters, while the test set is used for performance evaluation.

**Table 4:** **Additional set of data for testing and validation of MLP predictive models**.

**Exp. No. ANN**	**τ (min)**	**Ce (%)**	**ω (m/v)**	***t* (°**C**)**	**Y_obs_ (g/100 g d.e.)**	**MLP**
						**Y_pred_ (g/100 g d.e.)**
26^test^	10	30	1:12	36	9.900	9.808
27^test^	40	60	1:18	45	8.550	8.338
28^test^	50	70	1:20	30	11.790	11.642
29^test^	60	80	1:22	50	11.000	11.005
30^test^	80	90	1:25	60	14.630	14.717
31^validation^	100	96	1:5	25	16.530	16.705
32^validation^	110	100	1:20	30	21.300	21.805
33^validation^	120	100	1:25	40	24.830	24.808
34^validation^	60	40	1:20	70	7.210	7.204
35^validation^	45	80	1:21	22	13.500	13.697

*τ, extraction time; Ce, ethanol concentration; ω, solid-to-liquid ratio; t, extraction temperature; Y_obs_, observed value, Y_pred_, predicted value, d.e., dried extract*.

The training, validation and test data was within the limits of experiments. About 70% of the total number of experiments (35 experiments) was utilized for training of the neural network, while the rest was used for testing and validation, i.e., 15% each. The input variables of CCD (time extraction, ethanol concentration, solid-to-liquid radio and extraction temperature) were also used in the case of the neural network model. The content of amygdalin in the dried extract was defined as the output variable. The values of errors and correlation coefficients were calculated for all trained networks. The selection of optimal topology is selected based on these values. The optimal backpropagation neural network was trained to the minimum value of SSE, i.e., up to the 138th cycle. The structure of optimal MLP (4-3-1) network is presented in Supplementary Figure S.4. Exponential function was used as an activation function in the hidden layer with three neurons, while linear function was used in the output layer. The input and hidden layer have one more important element, i.e., bias. The bias allows to shift the activation function to the left or right, which may be critical for successful training. Generalization of the neural network was performed immediately after training process in order to analyze the adequacy of model for prediction of amygdalin content in the plum seeds extracts. The development of SSE in training and testing processes of the optimal neural network is also presented in Figure S.4. As it can be seen, the error development in both processes decreases by increasing the number of training cycles.

**Figure 1:**
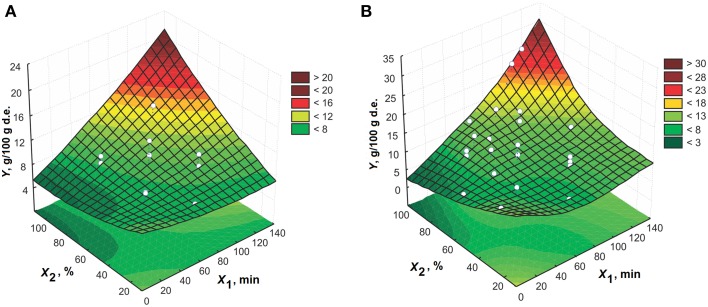
**The effect of extraction time and ethanol concentration on the amygdalin content in the extracts at 1:15 (m/v) and 50°C for model: (A) CCD and (B) MLP**.

**Figure 2:**
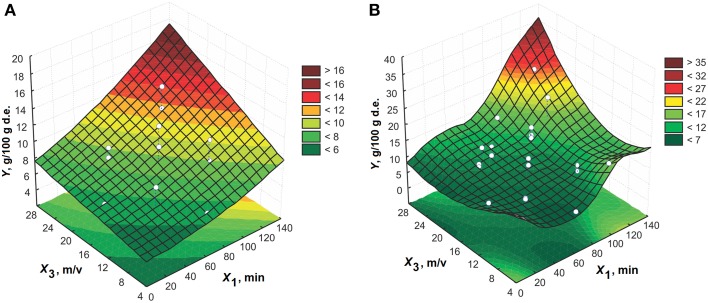
**The effect of extraction time and solid-to-liquid ratio on the amygdalin content in the extracts using 60% (v/v) ethanol at 50°C for model: (A) CCD and (B) MLP**.

**Figure 3:**
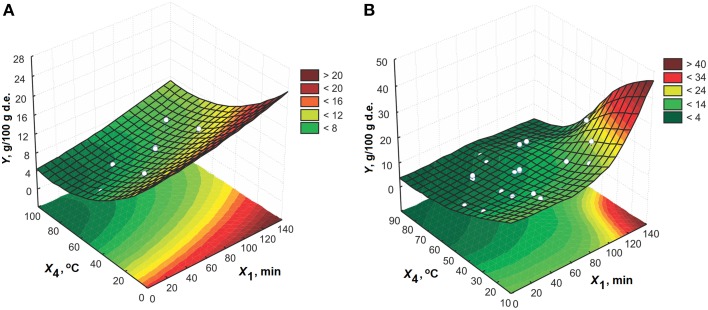
**The effect of extraction time and temperature on the amygdalin content in the extracts using 60% (v/v) ethanol at 1:15 (m/v) for model: (A) CCD and (B) MLP**.

**Figure 4:**
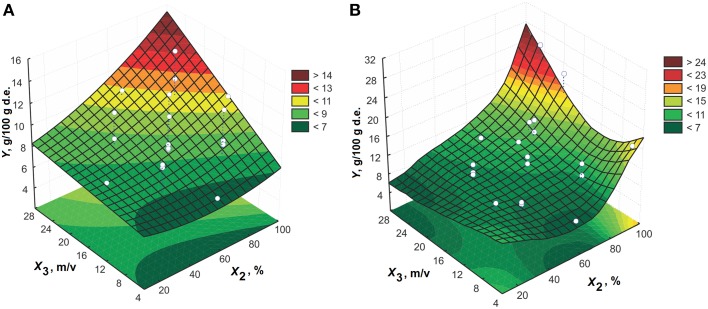
**The effect of ethanol concentration and solid-to-liquid ratio on the amygdalin content in the extracts for 65 min at 50°C for model: (A) CCD and (B) MLP**.

**Figure 5:**
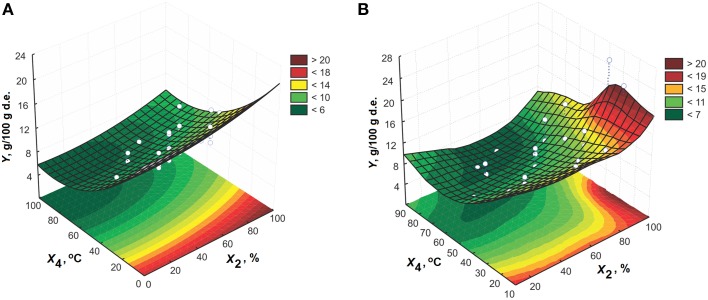
**The effect of ethanol concentration and extraction temperature on the amygdalin content in the extracts for 65 min at 1:15 (m/v) for model: (A) CCD and (B) MLP**.

**Figure 6:**
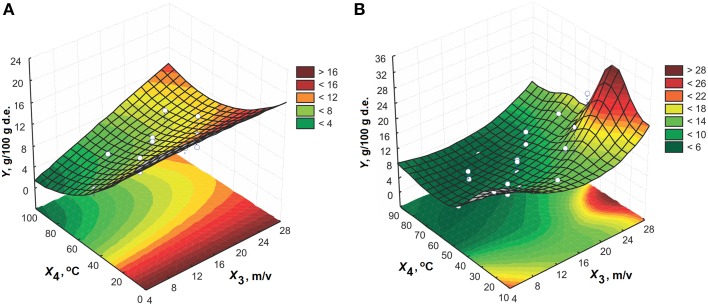
**The effect of solid-to-liquid ratio on the amygdalin content in the extracts for 65 min using 60% (v/v) ethanol for model: (A) CCD and (B) MLP**.

In Figures 1–**6**, the effects of all process variables on the amygdalin content in the extracts are presented for both models in the form of three-dimensional plots. Based on these plots, it is possible to better notice change in the response. The interactions between extraction time and ethanol concentration at solid-to-liquid ratio of 1:15 (m/v) and extraction temperature of 50°C for both CCD and MLP models are given in Figures 1A,B, respectively. The extraction time significantly impacts the amygdalin content in the extracts at higher levels of ethanol concentration. At lower levels of concentration, the influence of extraction time on response becomes meaningless, i.e., increasing the extraction time does not change the extraction efficiency. For short extraction times an increment in ethanol concentration leads to gradual increase in the response. The increase of ethanol concentration leads to enhancement of extraction efficiency in the case of longer extraction times. This is expected, because amygdalin is more exposed to the effect of emulsin enzyme action in the aqueous solutions. At these conditions, amygdalin is transformed to its inactive epimeric form, neoamygdalin (Koo et al., 2005; Lv et al., 2005). The reason for this behavior is probably due to inactivation of enzyme, so that amygdalin molecule remains unchanged. In addition to the fact that the solubility of amygdalin in water is better than in ethanol solution, the content of amygdalin in the extract obtained using water as the solvent is lower due to action of the enzyme.

The response surface of the effects of extraction time and solid-to-liquid ratio using 60% (v/v) ethanol at 50°C is shown in Figure 2. An increase in the process variables increases the amygdalin content in the extracts. The effect of extraction time on the response is significantly expressed at the higher solid-to-liquid ratios. The solid-to-liquid ratio has an important impact on the amygdalin content in the extracts for longer extraction times. The maximum point of three-dimensional plot is noticed at the solid-to-liquid ratio higher than 1:15 (m/v) and for extraction time longer than 100 min.

The plots that present the effect of extraction time and extraction temperature using 60% (v/v) ethanol at the solid-to-liquid ratio of 1:15 (m/v) are presented in Figure 3. The content of amygdalin in the extracts is higher at the lower temperatures. Thus, the high extraction temperature is not suitable for extraction of amygdalin from the plum seeds. The extraction time has a significant contribution to the increase of amygdalin content at lower temperatures, while this effect is negligible at higher temperatures. So the temperatures lower than 50°C are recommended for amygdalin extraction from this plant material.

The response surfaces of the effect of ethanol concentration and solid-to-liquid ratio on the performance of amygdalin extraction for extraction time of 65 min at 50°C are shown in Figure 4. The concentration of ethanol has a significant effect on the amygdalin content in the extracts at higher solid-to-liquid ratios. A similar behavior is noticed during analysis of the impact of solid-to-liquid ratio at ethanol concentrations higher than 80% (v/v).

The interaction between ethanol concentration and temperature for extraction time of 65 min and solid-to-liquid ratio of 1:15 (m/v) is presented in Figure 5. From these plots, it can be also concluded that the absolute ethanol is the solvent of choice. High temperatures have a negative effect on the amygdalin content in the extracts when using the solvents with high fraction of water. The decrease in the amygdalin content is probably the result of amygdalin epimerization in water mediums.

The response surfaces of the effect of solid-to-liquid ratio and temperature for extraction time of 65 min and 60% (v/v) ethanol are presented in Figure 6. The effect of solid-to-liquid ratio can be disregarded for temperatures higher than 30°C. The extraction efficiency increases by increasing solid-to-liquid ratio at higher temperatures.

The efficiency of the proposed models for prediction of the amygdalin content in the extracts was confirmed based on the values of errors and correlation coefficients. In the CCD model, a good agreement between the values of correlation coefficient indicates a validity of the proposed model. The values of errors and correlation coefficients for the MLP model in training, testing and validation processes are given in Table 5 in addition to the values for CCD model. The values of errors and correlation coefficients were calculated taking into account all data that were included into consideration for this model. Based on the lower values of errors in the case of MLP model, it can be concluded that the MLP model has a better performances compared with the CCD model.

**Table 5:**
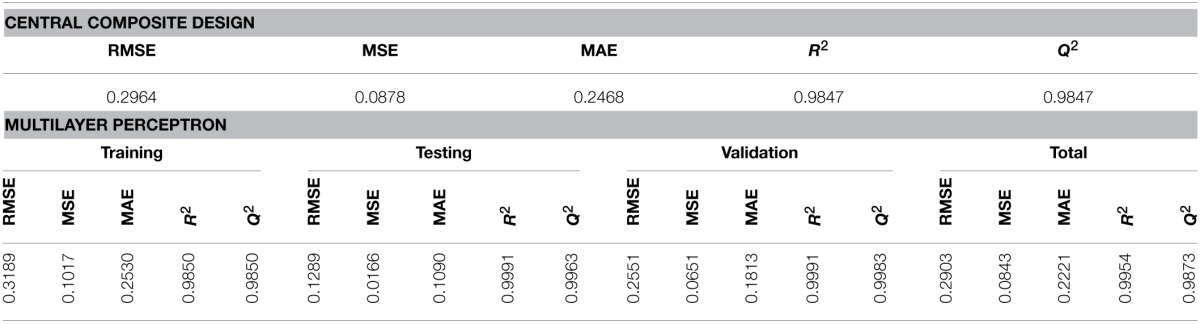
**The values of correlation coefficients and errors for CCD and MLP models**.

*RMSE, root mean square error; MSE, mean square error; MAE, mean absolute error*.

In order to find the maximum of the multidimensional curve the different optimization techniques were applied depending on the model. Actually, the optimal conditions for amygdalin extraction were obtained using numerical optimization method in the CCD model. The predicted value of the amygdalin content in the extract was 22.2 g per 100 g of the dried extract (d.e.) for extraction time of 116.3 min, 100% (v/v) ethanol, solid-to-liquid ratio of 1:24.3 (m/v) and extraction temperature of 32.1°C. There is a good agreement between the experimental (22.3 g/100 g d.e.) and predicted values for the amygdalin content in the extracts at these conditions, which indicates the adequacy of the proposed model.

The procedure of amygdalin extraction described by the MLP model was optimized using simplex method. After 400 iterations, the optimal conditions were achieved for the extraction time of 120 min, 100% (v/v) ethanol, solid-to-liquid ratio of 1:25 (m/v) and extraction temperature of 34.4°C. The predicted (25.42 g/100 g d.e.) and experimental (25.30 g/100 g d.e.) values of the amygdalin content in the extracts are almost identical.

The optimal extraction conditions for both models are almost identical despite to the fact that they were obtained using different optimization methods. In the case of the CCD model, the extraction time is shorter, solid-to-liquid ratio and extraction temperature lower compared with the conditions predicted by the MLP model. The amygdalin content in the extracts according to the MLP model is higher for about 3 g/100 g d.e. than the amygdalin content in the CCD model.

In the literature, the optimal conditions for amygdalin extraction from almond pollen were achieved after ultrasonic treatment for 30 min, using 100% (v/v) ethanol at solid-to-liquid ratio of 1:7 (m/v). In this case, the extraction time was 12 h (Zhang et al., 2007). For amygdalin extraction from the loquat (*Eriobotrya japonica*) under reflux, the optimal conditions were obtained using 65% (v/v) ethanol at solid-to-liquid ratio of 1:10 (m/v) and for temperature of 60°C after 45 min (Liu et al., 2012).

The optimal conditions found in the literature and those obtained in this study have similar ethanol concentration and extraction times despite to different plant materials. Unlike the reported procedures, in this study low temperature and high solid-to-liquid ratio are suggested for amygdalin extraction from plum seeds.

### Structural characterization of the isolated amygdalin

#### FTIR analysis

The isolated amygdalin from the extract of plum seeds is white powder without odor. Its purity of >90% was determined by comparison with available amygdalin standard using HPLC method. Based on IR spectroscopic method, the presence of functional groups of amygdalin was confirmed in the isolate. The IR spectra of isolated amygdalin and its standard are presented in Supplementary Figure S.5. In the structure of amygdalin, the primary and secondary hydroxyl groups from glucose part give the intensive and broad bands in the wavelength range of 3640–3630 cm^−1^. The bands at higher wavelength correspond to primary hydroxyl group, while the ones at lower wavelength correspond to secondary hydroxyl groups. The bands higher than 3000 cm^−1^ indicate C-H stretching vibrations of the aromatic ring, while the signal at ~2900 cm^−1^ indicates aliphatic C-H stretching vibrations. The stretching vibration of C-O bond caused the appearance of bands at 1100 cm^−1^. Also, the band at 2200 cm^−1^ can be noticed due to the presence of CN group in the structure of amygdalin. The amygdalin molecule contains cyclic ether bond, which gives the band at 1150 cm^−1^. The two bands at 1600 and 1400 cm^−1^ are the result of stretching vibration of C = C bond from benzene ring. The deformation C-H vibrations of aromatic ring caused the appearance of the band at 1450 cm^−1^. The out-of-plane C-H bending vibrations at 690 cm^−1^ indicate that benzene ring is monosubstituted. All these bands that correspond to the groups of amygdalin molecule without any new vibrations in the IR spectrum indicate a satisfactory purity of the isolated amygdalin.

#### UV analysis

Additional structural characterization of isolated amygdalin was performed using UV method. The samples were dissolved in methanol to the concentration of 50 μg cm^−3^ and analyzed by UV-VIS spectrophotometer (see Supplementary Figure S.6.). In UV spectrum, the absorption maximums at 184, 203, and 256 nm originate from π → π^*^ transition in aromatic system. The band at the lowest wavelength is the result of the allowed transition, while the other bands are due to the forbidden transition. The most intense absorption maximums are not of a high importance for identification of this chromophore, because it occurs in the spectra of the most substituted benzene at too small wavelengths–in the vacuum-ultraviolet. The secondary (forbidden) band is the result of ring deformation due to the vibration that distort the symmetry. This is the only reason of electron-vibration fine structure of the secondary band in the spectrum of benzene and other aromatics. In this study, the absorption maximums occurred at 215 nm, as well as between 250–254, 255–259, 261–265, and 267–271 nm. The absorption maximum of CN group was not noticed at around 300 nm due to its low intensity.

#### MS^2^ analysis

MS^2^ spectrum of the isolated amygdalin was recorded for the purpose of structural characterization. The mass spectrum of amygdalin recorded in the positive mode after the effect of collision energy of 12 eV is presented in Supplementary Figure S.7. The molecular peak can be noticed at *m/z* 480 as the result of formation of adduct with sodium [M+Na-H]^−^, while the protoned molecular ion at *m*/*z* 458 was not noticed. The peaks at m/z 453 and 363 were formed after losing the neutral fragments HCN and C_8_H_7_N (Ge et al., 2007). The most common product at *m*/*z* 347 and ionic product at *m*/*z* 374 are the results of losing mandelonitrile (C_8_H_7_NO) and C_7_H_8_N from the basic ion at *m*/*z* 480, respectively.

## Conclusion

This study compared the performance of the CCD and MLP models used for modeling of the procedure of amygdalin extraction from plum seeds. Both models provided similar optimal conditions for amygdalin extraction, but the conditions suggested by MLP are more preferable due to higher amount of desired compound. The amygdalin content in the extract predicted by MLP was 25.42 g/100 g d.e., while the experimental value was 25.30 g/100 g d.e. Also, the MLP model showed a higher accuracy in estimation. Thus, ANN could be a very powerful and flexible tool for modeling of the amygdalin extraction. Amygdalin is isolated from the ethanol extract of plum seeds in form of precipitate after addition of diethyl ether. The purity of isolated amygdalin compared with available amygdalin standard and determined by HPLC method was higher than 90%. The structure of isolated amygdalin was confirmed using FTIR, UV, MS^2^ methods.

### Conflict of interest statement

This article does not contain any studies with human or animals subjects. The authors declare that the research was conducted in the absence of any commercial or financial relationships that could be construed as a potential conflict of interest.

## Abbreviations

ANN: Artificial Neural Network
OVAT: One-Variable-at-a-Time
ANOVA: Analysis of Variance
CCD: Central Composite Design
MLP: Multilayer Perceptron
RSM: Response Surface Methodology
MAE: Mean Absolute Error
MSE: Mean Square Error
SS: Sum of Squares
MS: Mean Square
MS: Mass Spectrometry
FTIR: Fourier Transform Infrared Spectrometry
UV: Ultraviolet
RMSE: Root Mean Square Error.
