# Supplementary material for: Optimization of technological procedure for amygdalin isolation from plum seeds (Pruni domesticae semen)
Source: Front Plant Sci. 2015 Apr 28;6:276. doi: 10.3389/fpls.2015.00276 (PMC4411975; doi:10.3389/fpls.2015.00276)
Supplement: Supplementary file 1 [file DataSheet1.DOC]

**SUPPORTING INFORMATION AVAILABLE**


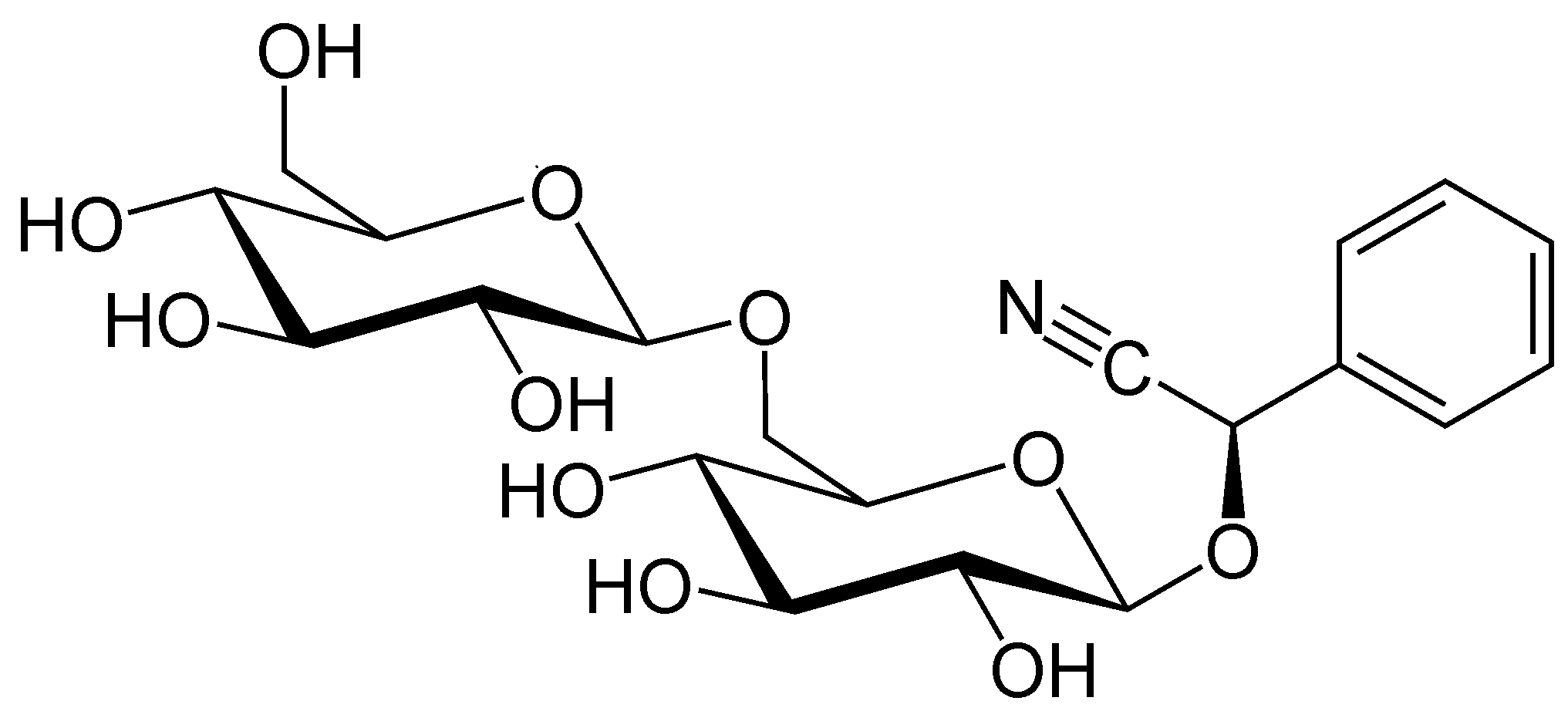


**Figure S.1.** Chemical structure of amygdalin

**
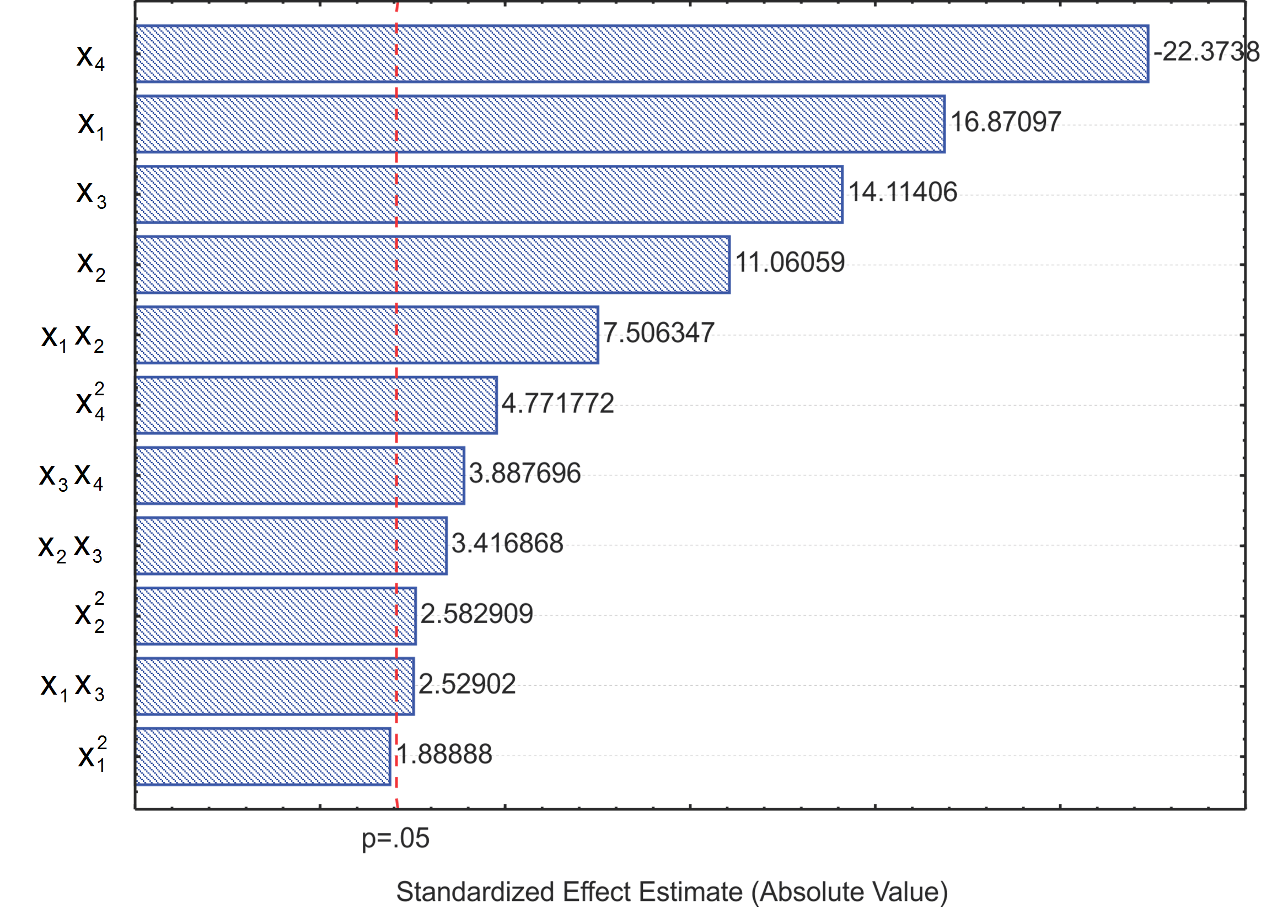
**

**Figure S.2.** Pareto chart of standardized effects

**
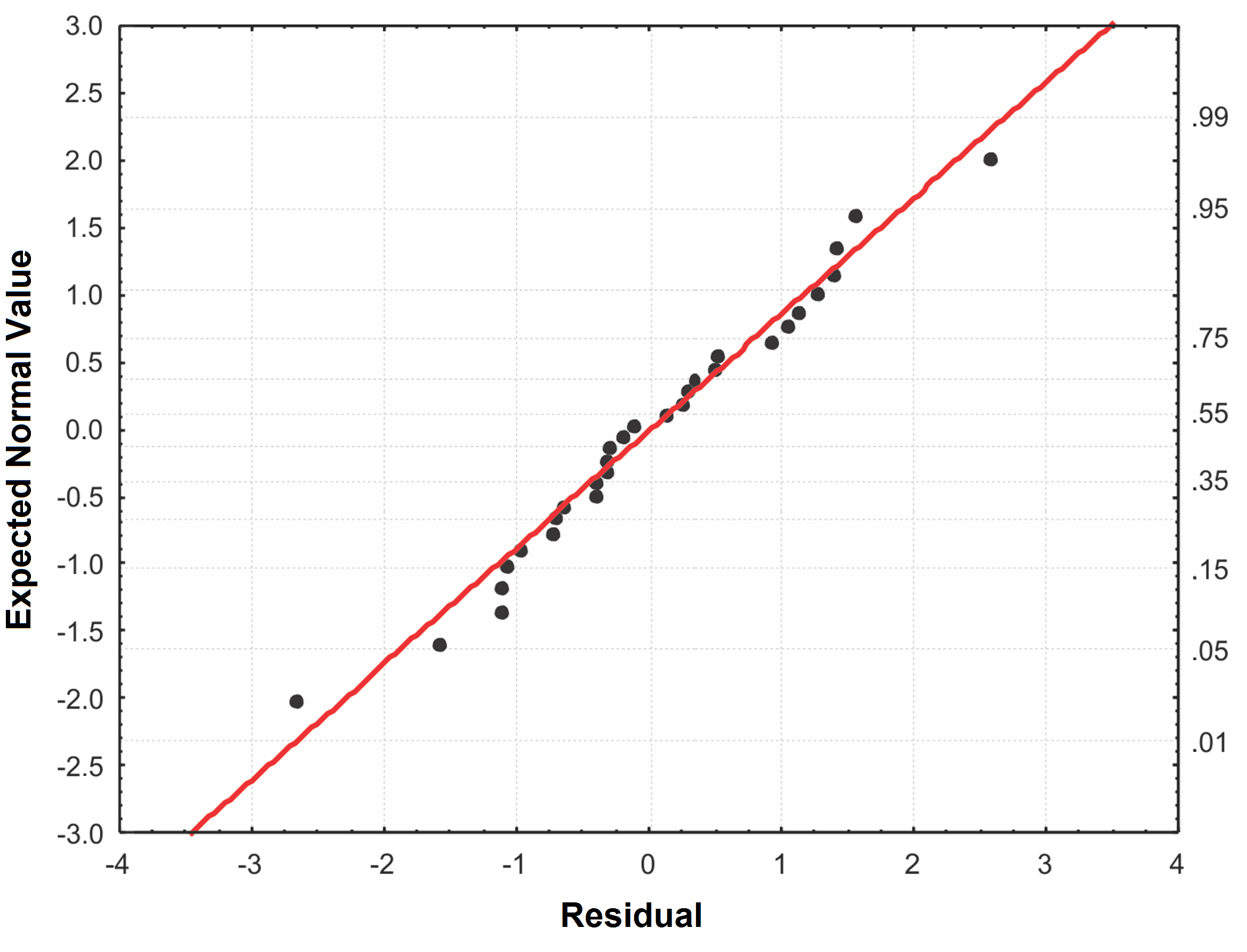
**

**Figure S.3.** Normal probability plot of standardized residuals


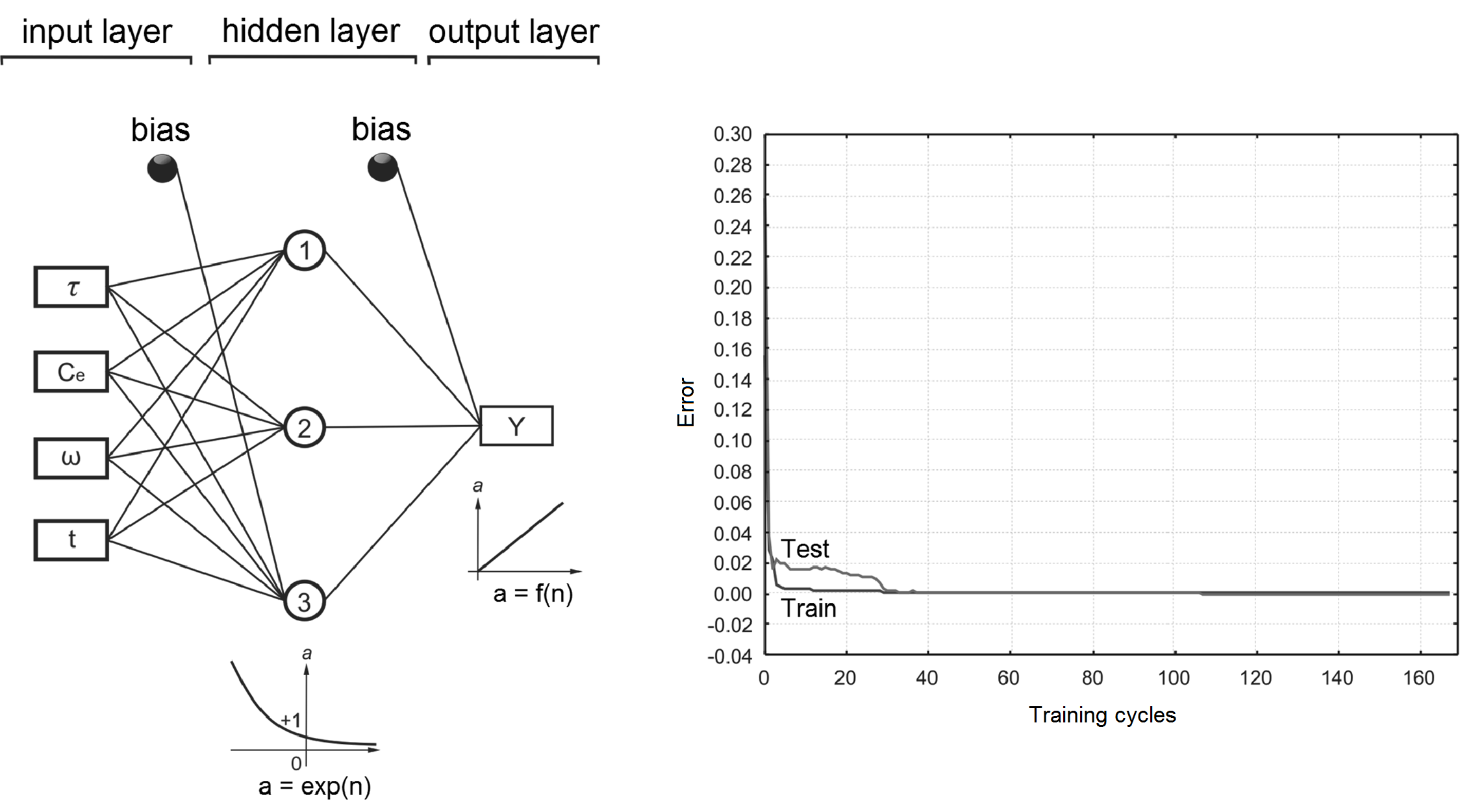


**Figure S.4.** Optimal neural network and plot of error development in training and testing processes


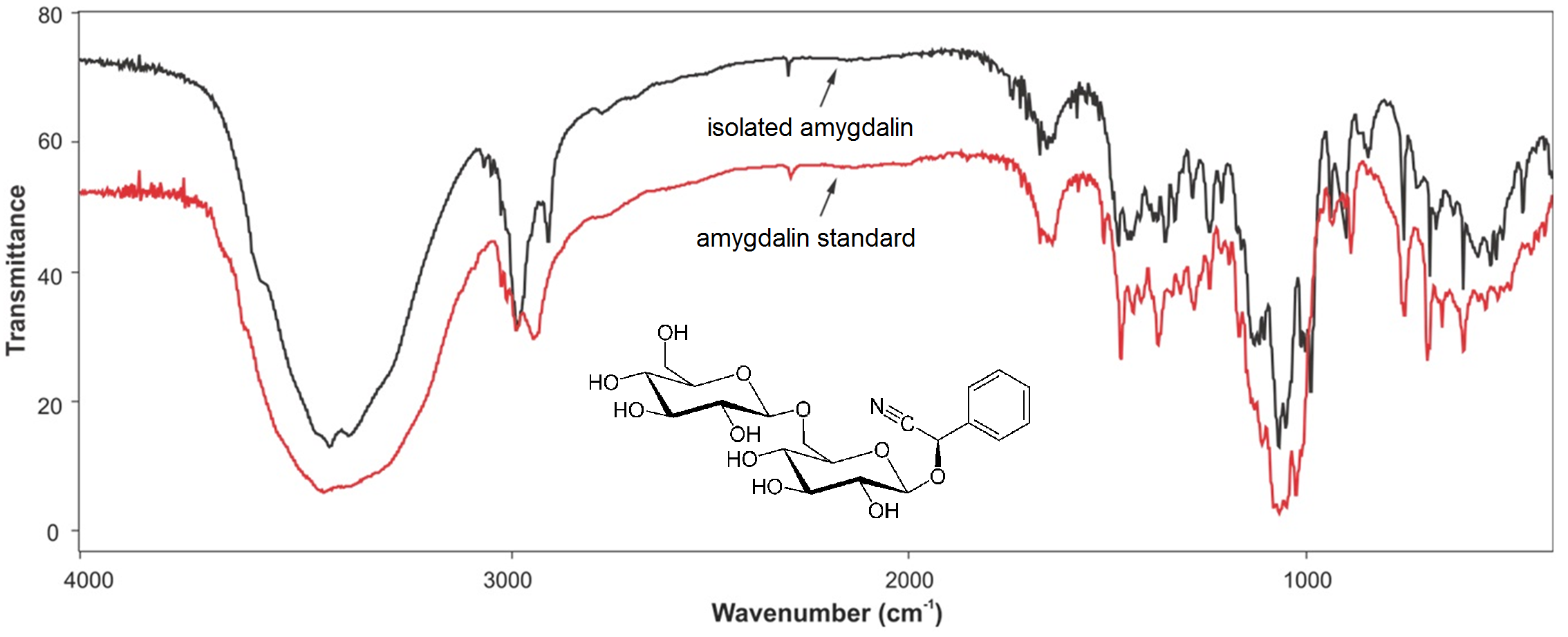


**Figure S.5.** IR spectra of isolated amygdalin and amygdalin standard


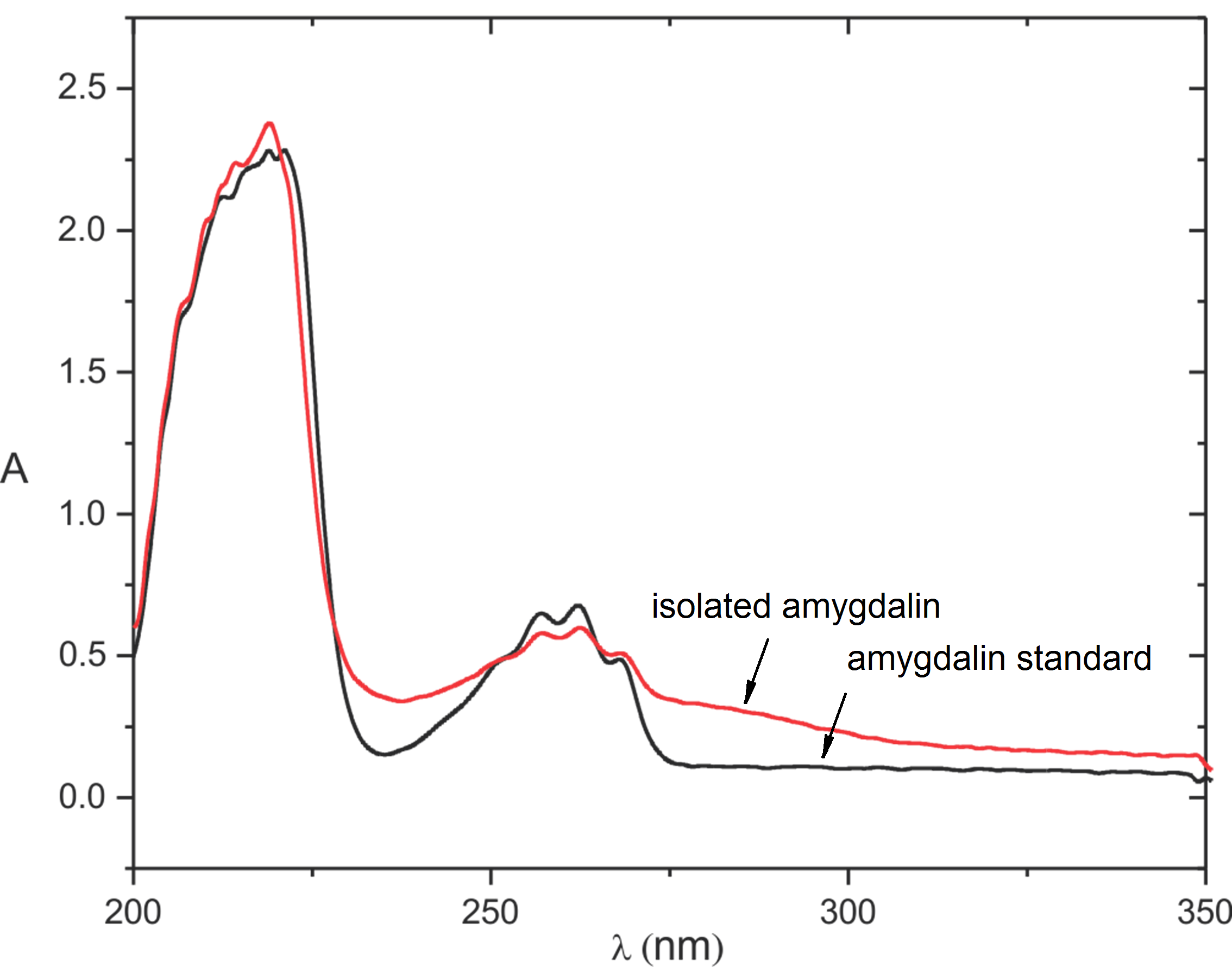


**Figure S.6.** UV spectra of isolated amygdalin and amygdalin standard (50 mg dm-3) in methanol


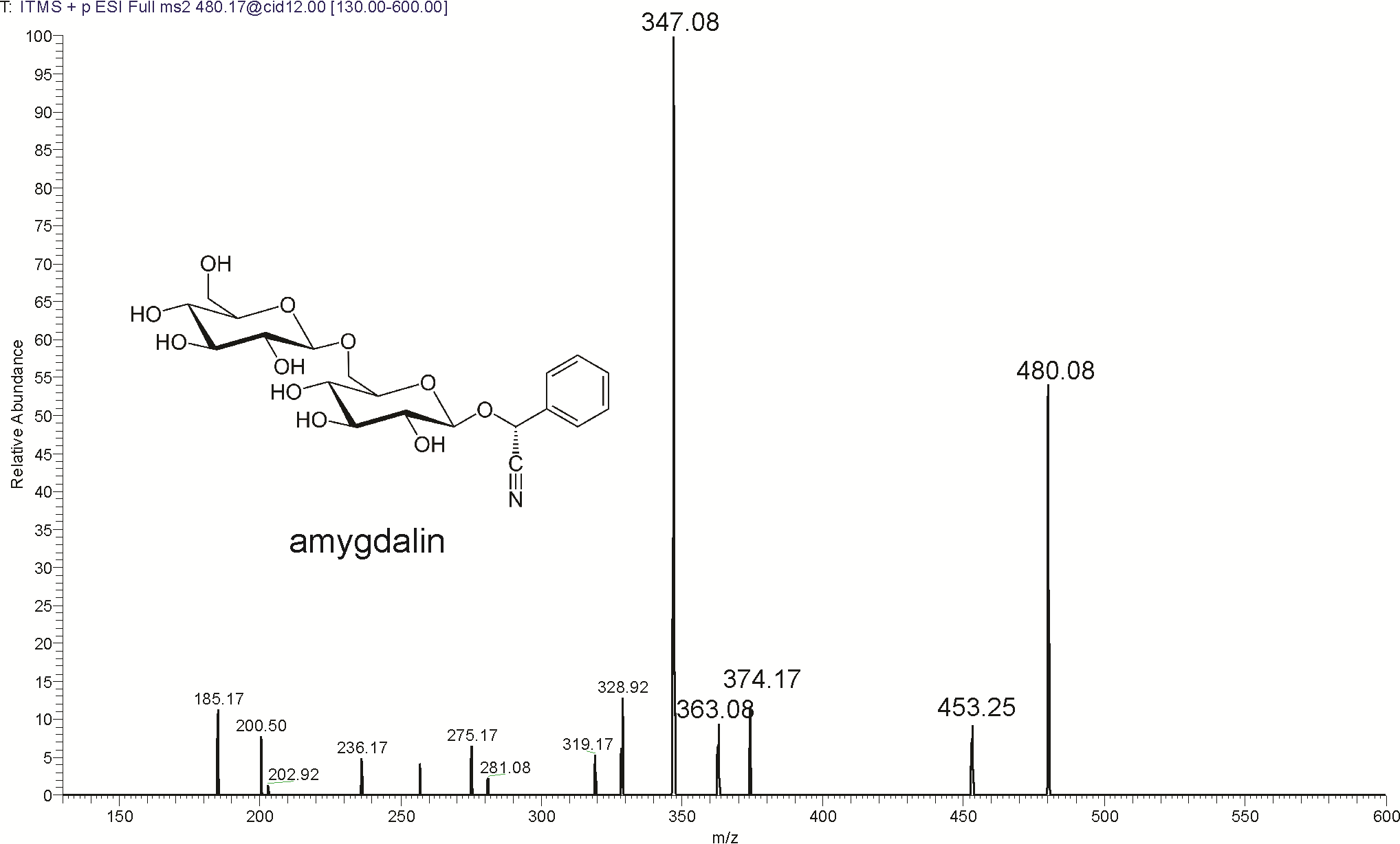


**Figure S.7.** MS2 spectrum of isolated amygdalin recorded in the positive mode
